# Special Section Guest Editorial: Biomedical Imaging and Sensing II

**DOI:** 10.1117/1.JBO.25.3.032001

**Published:** 2020-02-26

**Authors:** Toyohiko Yatagai, Osamu Matoba, Yoshihisa Aizu, Yasuhiro Awatsuji, Yuan Luo

**Affiliations:** a Utsunomiya University, Utsunomiya, Japan; b Kobe University, Kobe, Japan; c Muroran Institute of Technology, Muroran, Japan; d Kyoto Institute of Technology, Kyoto, Japan; e National Taiwan University, Taipei, Taiwan

## Abstract

Guest editors introduce the special section of Journal of Biomedical Optics Volume 25, Issue 3, entitled “Biomedical Imaging and Sensing II,” a collection of papers related to the topics of the conference “Biomedical Imaging and Sensing Conference 2019” (BISC’19), which was held in April 2019, in Yokohama, Japan.

This special section of JBO Volume 25, Issue 3, entitled “Biomedical Imaging and Sensing II,” provides a collection of papers related to the topics of the conference “Biomedical Imaging and Sensing Conference 2019” (BISC ‘19), which was held from April 24 to 26 in Yokohama, Japan. This conference provides an international forum for reporting recent progress in imaging and sensing technologies in the fields of biology and medicine, as well as related areas.

In biomedical optics and photonics, optical tools are employed for the understanding and treatment of diseases, from the cellular level to macroscopic applications. At the cellular level, highly precise laser applications allow the manipulation, operation, or stimulation of cells, even in living organisms or animals. Optical microscopy has been revolutionized by increased understanding of the different markers and their switching behavior. Marker-free microscopy technologies, like CARS (coherent anti-Stokes Raman scattering), SHG- (second-harmonic-generation) or THG- (third-harmonic-generation) microscopy, optical coherence tomography (OCT), and DHM (digital holographic microscopy), are spreading into multiple biological and clinical imaging applications. OCT is continuously broadening its clinical applicability by becoming even higher resolution, higher speed, and more compact. In the broader field of optics and photonics, biomedical imaging and sensing are the most quickly progressing and expanding areas. Techniques developed in these areas could greatly advance physical, engineering, and biological knowledge, as well as optics and photonics technology.

This conference includes basic research at the cellular level through clinical applications of various optical technologies. Topics covered include medical and biological imaging instrumentation and techniques, advanced microscopy, advanced endoscopy, super resolution, computational imaging, adaptive optics, structured illumination, OCT, digital holography, quantitative phase imaging, diffuse spectroscopy and tomography, photoacoustic imaging, multimodal imaging and sensing, optical biopsy, multispectral imaging and sensing, spectroscopic imaging and sensing, scattering imaging, fluorescence imaging, molecular imaging, terahertz sensing, imaging and sensing techniques for biomedicine, optical fibers and sensors for biomedicine, and multimodal optical diagnostic systems. Recently, computational imaging such as light field imaging, digital holography, and compressive sensing is expected to open new imaging techniques to improve image quality, obtain multimodal physical parameters, and apply the weak light condition using a photon-counting device.

This special section includes interesting articles on OCT endoscopy, multimodal digital holographic imaging, computational fluorescence 3D imaging, and laser speckle contrast imaging for medical applications. We believe that this special section on biomedical imaging and sensing will serve to advance biomedical imaging and sensing techniques.

